# Coexistence of Virulence Factors and Efflux Pump Genes in Clinical Isolates of *Pseudomonas aeruginosa*: Analysis of Biofilm-Forming Strains from Iran

**DOI:** 10.1155/2021/5557361

**Published:** 2021-05-21

**Authors:** Shahram Shahraki Zahedani, Hamed Tahmasebi, Mojdeh Jahantigh

**Affiliations:** ^1^Department of Microbiology, Zahedan University of Medical Sciences, Zahedan, Iran; ^2^School of Medicine, Shahroud University of Medical Sciences, Shahroud, Iran

## Abstract

**Background:**

Biofilm formation and efflux pumps (EPs) correlation play a critical role in the pathogenicity and antibiotic resistance of *Pseudomonas aeruginosa*. In this study, biofilm formation and EP's collaborative role in clinical isolates of *P. aeruginosa* infection were investigated.

**Methods:**

Eighty-six (86) *P. aeruginosa* isolates were collected from different clinical specimens and were confirmed using different biochemical tests. The formation of biofilm was investigated by using a crystal violet assay. Also, EP genes were identified by the PCR method.

**Results:**

Based on the results, gentamicin-resistant (*n* = 50, 66.29%) and ciprofloxacin-resistant (*n* = 61, 69.66%) strains were the most frequent and colistin (*n* = 1, 1.12%) and ceftazidime (*n* = 12, 7.86%) resistant strains were the least prevalent. Furthermore, 22 isolates (31.42%) were MDR, and 11 isolates (12.35%) were XDR strains. Also, 19 isolates (22.47%) were classified as strong biofilm, 29 *isolates* (21.34%) as moderate biofilm, and 3 (11.23%) isolates as weak biofilm producers. The distribution of the EP genes was as follows: *mexA* (*n* = 44, 34.83%), *mexB* (*n* = 33, 32.58%), *oprM* (*n* = 59, 29.21%), *oprD* (*n* = 61, 30.33%), *tetA* (*n* = 22, 25.58%), *tetR* (*n* = 19, 22.09%), and *emrE* (*n* = 21, 24.41%). However, there was a strong significant association between biofilm formation and EPs in *P. aeruginosa. Conclusions*. In this study, we suggested that the presence of a multidrug resistance efflux pump, MexEF-OprN, significantly reduced *P. aeruginosa* pathogenicity. In contrast, the presence of the MexAB-OprM and MexCD-OprJ pumps did not affect virulence.

## 1. Introduction

*Pseudomonas aeruginosa* (*P. aeruginosa*) is a paradigm of an opportunistic clinical pathogen with innate resistance to many antibiotics. In humans, *P. aeruginosa* is mainly of great concern in severe burns, cancer, and AIDS patients, as well as those people who are immunosuppressive [1, 2]. Other essential infections caused by the organism are pneumonia, endocarditis, endophthalmitis, meningitis, septicemia, and conjunctivitis. The frequency of *P. aeruginosa* is high in surgical and burn wound infections [3].

In general, *P. aeruginosa* is naturally less susceptible than other Gram-negative bacilli to many antibiotics. Multidrug-resistant (MDR) *P. aeruginosa* strains are of particular concern and pose a significant clinical challenge [4, 5]. Separate MDR strains are because there is a synergy between the multidrug efflux system and low outer membrane permeability. One of the organism's crucial inherent characteristics is resistance to various antibiotics and disinfectants, mainly due to the multidrug efflux system and low outer membrane permeability [6, 7].

Efflux pumps (EPs) are membrane transporter proteins representing a significant component of the intrinsic and acquired antibiotic resistance mechanisms in *P. aeruginosa* [8]. This organism is intrinsically resistant to many structurally unrelated antimicrobial agents because of the low permeability of its outer membrane and the constitutive expression of various EPs with broad substrate specificity [7, 9]. However, four well-known genetically different efflux systems were also had been identified as responsible for multidrug resistance (MDR) in *P. aeruginosa,* namely, MexAB-DprM, MexCD-OprJ, MexEF-OprN, and MexXY-OprM. Each pump has a preferential set of antimicrobial agent substrates. The EP genes are present in all strains, but they are not expressed at high levels. However, the increased expression can result from a mutation in regulatory genes such as *mexR*, which controls the mexAB-OprM genes [2, 10].

Various investigations suggest a correlation between EPs and biofilm formation [6]. However, the direct effect of EPs on bacterial pathogenicity and virulence is unclear. Any defect in EP activity can impair biofilm formation. Therefore, inhibiting any of the efflux activity by inhibitors can reduce biofilm formation [11]. *P. aeruginosa* is an opportunistic pathogen associated with chronic infections. It is one of the leading causes of hospital- and community-acquired infections. Virulent *P. aeruginosa* is frequently life threatening, and the emergence of multidrug-resistant isolates often presents challenges to treat the patients. The interplay between both resistance and virulence is always considered together. The extent of adaptation of bacteria to many adverse environments is the primary concern among health-care centers. So, the analysis of these essential bacterial characteristics is crucial to management strategies. However, biofilm growth on medical devices and tissue surfaces can lead to biofilm formation and increase the risk of wound and respiratory infections [12, 13]. Also, EPs and biofilm in *P. aeruginosa* are essential for both clinical and environmental isolates to tolerate desiccation [11].

Therefore, this study is conducted to understand other roles and relationships of EP types with biofilm formation of different clinical isolates of *P. aeruginosa* in the south of Iran.

## 2. Materials and Methods

### 2.1. Study Design and Collection of Isolates

In this descriptive-analytical study, 510 different clinical specimens, including blood, urine, wound, burn wound, catheter, and abscess, were collected from patients admitted to teaching hospitals in Zahedan, Iran, from September 2018 to May 2019. The isolates were then streaked on Luria Bertani (LB) and McConkey agar plates on reaching the laboratory. Their identities were reconfirmed by Gram staining, motility testing, and biochemical reactions, essentially described by Tahmasebi et al. [1]. However, 86 *P. aeruginosa* isolates were collected. All ethical standards have been respected in preparation for the submitted article, No. IR.ZAMUS.REC.1396.140.

### 2.2. Determination of the MIC Pattern

An antibiotic susceptibility test by the Disc Diffusion Test (DDT) was carried out for all the biochemically confirmed isolates of *P. aeruginosa*. However, the isolates were categorized as sensitive, resistant, or intermediate to each antibiotic by measuring the respective zone of inhibition and were finally interpreted following the CLSI guidelines. The DDT is based on using the disc, as meropenem, imipenem, cefepime, ceftazidime, gentamicin, amikacin, ciprofloxacin, colistin, aztreonam, and trimethoprim/sulfamethoxazole (MAST, UK). E-test strips (Liofilchem, Italy) were used for determining colistin-resistant strains.

### 2.3. Screening of Biofilm Producer Strains

The capacity to form biofilms was assayed in microtiter plates virtually, and the crystal violet method (CVM) was described by Azeredo et al. [14]. In this case, *P. aeruginosa ATCC* 19606 was used as the positive control, and the culture medium was used as the negative control.

### 2.4. DNA Extraction

DNA was extracted from all the phenotypically confirmed *P. aeruginosa* isolates by the boiling method according to Tahmasebi et al.'s study [15].

### 2.5. Detection of EP Genes

Specific primers (Table 1) were used to amplify EP genes. The PCR reaction was performed in a total volume of 25 *µ*L. Reactions were contained 1 *µ*L of DNA template, 12 *µ*L Master Mix (Fermentas, Waltham, Massachusetts, United States), 1 *µ*M of each primer, and 10 *µ*L deionized water (Sigma-Aldrich, USA). PCR assays were performed (based on Table 1) in a Bio-Rad MJ Mini thermal cycler (T100cyclerBio-Rad, Hemel Hempstead, UK) with a heated lid. On completion of the reaction, tubes with PCR products were held at 4°C. Further analysis and confirmation were carried out by performing analytical agarose gel electrophoresis (1% agarose gel for 95 min at 85 V) [16].

### 2.6. Statistical Analysis

Data were statistically analyzed using GraphPad Prism software version 8 (GraphPad Software, Inc., CA, US) and an appropriate statistical test, i.e., either Student's*t*-test, Wilcoxon test, chi-square test, or one-way ANOVA.

## 3. Results

Among 86 isolates of *P. aeruginosa*, 19 isolates (22.93%) from male patients and 67 isolates (72.04%) from female patients were collected. As shown in Table 2, 15 were from urine (17.44%), 21 from the wound (24.41 %), 11 from indwelling medical devices (12.79%), and 29 from the blood (33.72%). Samples were collected from patients hospitalized in the maternity unit (*n* = 6), pediatrics (*n* = 14), internal (*n* = 13), emergency (*n* = 17), neurology (*n* = 7), intensive care unit (ICU) (*n* = 15), and burns unit (*n* = 14).

### 3.1. Antibiotic Resistance Pattern

**Figure 1:**
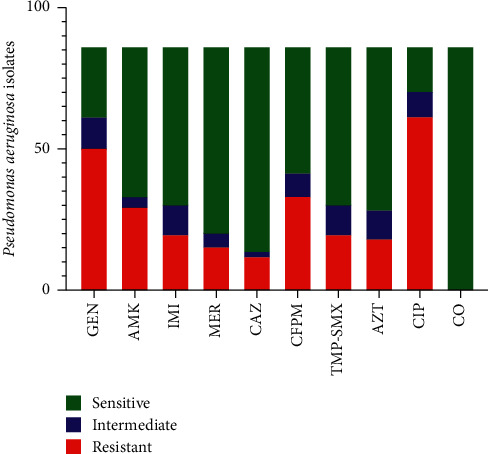
Antibiotic resistance pattern in clinical isolates of *P. aeruginosa*. IMI: imipenem; MER: meropenem; GEN: gentamycin; CIP: ciprofloxacin; ATM: aztreonam; CPE: cefepime; C: colistin; AMK: amikacin; TMP/SMX: trimethoprim/sulfamethoxazole.

Resistance to ciprofloxacin (70.93%) and gentamicin (58.13%) was the most frequent and followed by cefepime (32.20%), amikacin (33.72%), and aztreonam (19.76%). Resistance to ceftazidime (13.95%) was the least detected among isolates. Also, 22 MDR isolates (31.42%) and 11 XDR isolates (15.71%) were reported (Figure 1).

### 3.2. Biofilm Production

The results for biofilm formation are shown in Table 2. In 86 of *P. aeruginosa*, 51 isolates (59.30%) were considered biofilm producers, and 35 isolates (40.96%) lacked biofilms. Also, 19 isolates (22.09%) were classified as strong biofilm, 29 isolates (33.72%) as moderate biofilm, and 3 (3.48%) isolates as weak biofilm producers. However, significant correlations between the biofilm formation and the prevalence of antibiotic-resistant bacteria have been observed. Decreases in antibiotic resistance prevalence in biofilm-forming strains were statistically significant except for colistin and aztreonam (*p* > 0.05).

### 3.3. Prevalence of EP Genes

**Figure 2:**
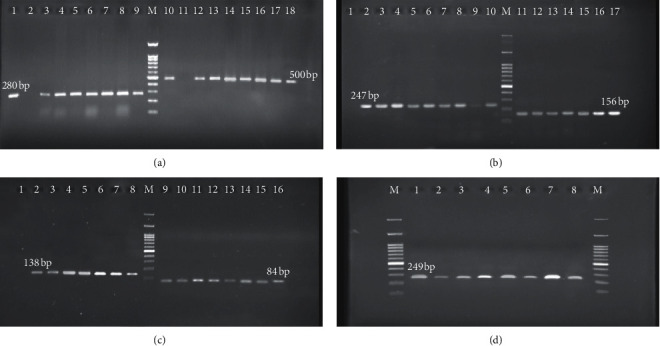
The amplification and gel electrophoresis agarose 2% of EP genes in *P. aeruginosa*. (a) *mexA* with 503 bp, *mexB* with 280 bp; well 1: positive control, wells 3 to 9: positive strains with *mexB*; well 10: positive control, and wells 11 to 18: positive strains with *mexA*. (b) *oprM* with 247 bp, *oprD* with 156 bp; well 2: positive control, wells 3 to 10: positive strains with *oprM*; well 11: positive control, and wells 12 to 18: positive strains with *oprD*. (c) *tetA* with 138 bp, *tetR* with 84 bp; well 1: positive control, wells 2 to 7: positive strains with *tetA*; well 7: positive control, and wells 8 to 14: positive strains with *tetR*. (d) *emrE* with 249 bp; well 1: positive control and wells 2 to 8: positive strains with *emrE.* M Ladder 100 bp.

**Figure 3:**
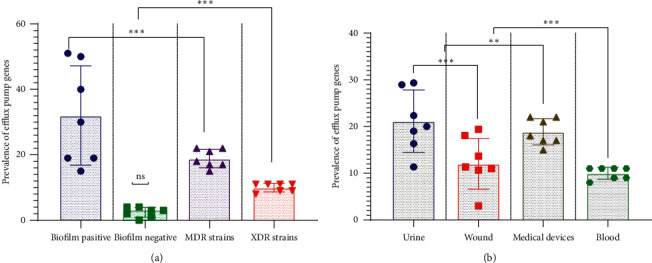
Association between efflux pump genes, antibiotic resistance, biofilm formation, and clinical samples in *P. aeruginosa* isolates. (a) Association between efflux pump genes, antibiotic resistance, and biofilm formation. (b) Association between efflux pump genes and clinical samples. Each dataset was analyzed using Student's *t*-test and the two-way ANOVA and was presented as Mean + SEM. ^*∗*^*p* value <0.05; ^*∗*^^*∗*^*p* value <0.01; ^*∗*^^*∗*^^*∗*^*p* value <0.001; and ^*∗*^^*∗*^^*∗*^^*∗*^^*∗*^*p* value <0.0001. ns: nonsense.

According to Figure 2, 59 isolates (68.60%) were *oprM* and 61 isolates (64%) were *oprD*, followed by 44 isolates (67.44%), 33 isolates (38.37%), 22 isolates (25.58%), 21 isolates (24.41%), and 19 isolates (22.09%) detected as *mexA*, *mexB*, *tetA*, *ermE*, and *tetR*, respectively (Table 2) (Figure 2). However, Figure 3 and Table 3 indicates the statistical association between antibiotic resistance and EPs genes. Also, a strong relationship was observed between biofilm formation and EP genes (*p* ≤ 0.05). The *MexA*, *mexB*, *oprM*, *tetA,* and *oprD* genes were presented significantly more frequently among MDR and XDR isolates and biofilm‐forming isolates. In contrast, the *tetR* and *emrE* genes were significantly more frequent among antibiotic-sensitive isolates. There was no statistical association between some of the antibiotics, such as colistin and EP genes (*p* > 0.05).

## 4. Discussion

This study showed that the ceftazidime- and colistin-susceptible strains were the most frequent, as is described in Figure 1. In the present study, 13.95% of ceftazidime-resistant strains were detected from *P. aeruginosa* isolates. Also, colistin-resistant strains were not detected in isolates. Dehbashi et al. and Rodulfo et al. reported similar results [1, 17]. However, according to Figure 1, a high rate of gentamycin resistance (58.13%) and ciprofloxacin resistance (70.93%) was observed in isolates. These results agree with the study conducted by Kuti et al. and Ijaz et al. [18, 19].

The ability of *P. aeruginosa* to resist desiccation and form biofilms allows it to survive for long periods on abiotic surfaces. In the current study, Table 2 displays that 22.47% of isolates were classified as strong biofilm and 21.34% as moderate biofilm. The MDR strains in 95% of strong biofilm isolates and 89/47% of moderate biofilm isolates were reported. Furthermore, XDR strains were detected in 82.44% of strong biofilm and 78.94% of moderate biofilm. Yang et al. examined the effects of biofilm formation on imipenem efficacy. However, they reported that imipenem efficacy was significantly reduced in aged biofilms [20]. Rahimi et al. found that biofilm-producing *P. aeruginosa* had comparatively higher resistance to amikacin than non-biofilm-forming strains. Also, MDR/XDR strains show a strong relationship between biofilm formation and antibiotic resistance [21].

Investigating the prevalence of RND-type, MFS-type, and SMR-type EP encoding genes, we report that these efflux systems are widely distributed in *P. aeruginosa*. However, the *mexA*, *mexB*, *oprM*, and *oprD* genes of RND-EP were present in 51.62%, 38.37%, 68.60%, and 70.93% tested isolates, respectively. Furthermore, the distribution of the SMR-EP gene was observed in 24.42% of *P. aeruginosa*. However, *tetA* and *tetR* MFS-EP genes were detected in 25.58% and 22.09% of isolates, respectively. Murugan et al. reported the same results and demonstrated that RND-EP genes were more abundant in MDR/XDR strains of *P. aeruginosa* than other EP genes [22].

However, the EP can also alter the pathogenicity of *P. aeruginosa* and play an important role in biofilm formation. As shown in Table 2, all EP families were characterized by biofilm producer strains. To our knowledge, this is the first report describing the distribution of three EP families in Iran and reporting their associated with biofilm formation in *P. aeruginosa*. Alav et al. [6] and Rumbo et al. [23] found that isolates with the EP gene had a greater capacity for biofilm formation than strains that lacked the gene. However, Rampioni et al. confirm the relationship between EPs and biofilm formation in *P. aeruginosa*; it should be noted that this association depends on many factors [24].

Based on Table 2, the strains with efflux systems were the most frequent in wound and urine specimens. Nevertheless, as shown in Table 3 and Figure 3, we found a strong association between the biofilm formation and its EP gene profile. These findings indicated that EPs play an essential role in biofilm formation and the rate of antibiotic resistance in *P. aeruginosa*. There was also a significant relationship between EPs' family and biofilm formation in *P. aeruginosa*. In agreement with these findings, Horna et al. and Shigemura et al. reported a significant relationship between the biofilm formation, EPs, and pathogenicity of *P. aeruginosa* isolated from wound and urine. They also stated that strains with EPs are resistant to treatment and have a more substantial role in causing urinary tract infections [25, 26]. In the current study, there was a significant relationship between EP families and biofilm formation. The frequency of RDN-EP and MFS-EP was higher in biofilm producer strains. This observation is similar to Minagawa et al.'s finding, which suggested that RND-EP plays a more important role in *P. aeruginosa* [27]. Soto showed that strains with EPs were more prone to biofilm formation. He stated a significant relationship between biofilm formation and EPs in Gram-negative bacteria [28].

Moreover, bacteria with EPs are more pathogenic than bacteria without EPs and play a critical role in wound and urinary tract infections. Hence, the type of clinical samples is an essential factor in this correlation [29, 30]. However, biofilm growth is associated with an increased level of mutations and quorum-sensing-regulated mechanisms. Conventional resistance mechanisms such as chromosomal *β*-lactamase, upregulated efflux pumps, and mutations in antibiotic target molecules in bacteria also contribute to biofilms' survival [31, 32].

Moreover, the multiple resistance to the most commonly used antibiotics is quite common in *P. aeruginosa* due to the possession of a high number of virulence factors [33]. A high antibiotic resistance level is attributable to multidrug efflux pumps' concerted action with a chromosomally encoded antibiotic resistance gene and the low permeability of bacterial cellular envelopes and biofilm formation phenomenon [34].

In summary, our study supports the idea of a relationship between EPs and biofilm formations. Biofilm-forming capacity in *P. aeruginosa* with EPs presumes a vital part in the host-pathogen communications and medical-device-related infection. Also, EPs constitute a significant threat in the clinical wound by acting as reservoirs of multidrug-resistant pathogenic bacteria. *P. aeruginosa* can become more resistant due to environmental conditions and different physiological states such as biofilms. It is necessary to pay attention to the slight increases in resistance observed in the clinic because this probably indicates the emergence of adaptative resistance during the wound infection and possible treatment troubles. Thus, the resistance status at arrival should ideally be controlled as a potential confounding variable for the association between resistance at the biofilm-forming strains and exposure to antimicrobial drugs. These results suggest that *P. aeruginosa* has a great tendency to biofilm formation in wound infections, which causes an increase in antibiotic resistance.

## Figures and Tables

**Table 1:** Oligonucleotide sequences used in this study and thermal cycling conditions.

Gene	Sequence of primers	Thermal cycles	Product size (bp)	Ref
*mexA*	F: CTCGACCCGATCTACGTC	95°C/5 min; (95°C/1 min, 56°C/30sec, 72°C/45sec) X30; 72°C/5 min	503	[8]
	R: GTCTTCACCTCGACACCC			

*mexB*	F: TGTCGAAGTTTTTCATTGAG	95°C/5 min; (95°C/1 min, 58°C/45sec, 72°C/45sec) X35; 72°C/5 min	280	[8]
	R: AAGGTCAC GGTGATGGT			

*oprM*	F: GATCCCCGACTACCAGCGCCCCG	95°C/5 min; (95°C/1 min, 57°C/1 min, 72°C/45sec) X30; 72°C/5 min	247	[8]
	R: ATGCGGTACTGCGCCCGGAAGGC			

*oprD*	F: ATCTACCGCACAAACGATGAG	95°C/5 min; (95°C/1 min, 59°C/45sec, 72°C/1 min) X30; 72°C/5 min	156	[8]
	R: GCCGAAGCCGATATAATCAAAC			

*tetA*	F: AAGAATCCGCGCGTTCAATCG	95°C/7 min; (95°C/1 min, 55°C/1 min, 72°C/50sec) X35; 72°C/10 min	138	[15]
	R: GCCCGGCACCGGCATAAT			

*tetR*	F: CCGAATGCGTATGATTCTCC	95°C/10 min; (95°C/1 min, 57°C/30sec, 72°C/1 min) X30; 72°C/10 min	84	[15]
	R: CGCTTTACTGGCACTTCAGC			

*emrE*	F: CCGCCATGACCAACTATCTC	95°C/5 min; (95°C/1 min, 58°C/1 min, 72°C/1 min) X30; 72°C/5 min	249	[6]
	R: GCTGGCCGTAGACGAACATC			

**Table 2:** The biofilm-forming capacity of *P. aeruginosa* and percentages of their efflux pump genes related to antibiotics.

Biofilm	Efflux pump genes							IMI	MER	GEN	CIP	ATM	CPE	CAZ	C	AMK	TMP/SMX	MDR	XDR
	RND types			MFS types			SMR types												
	*MexA*	*mexB*	*oprM*	*oprD*	*tetA*	*tetR*	*emrE*												
Strong (*n* = 19, 33.72%)	12	19	17	19	11	8	7	11	9	19	16	7	6	9	1	16	7	14	9
Moderate (*n* = 29, 33.72%)	19	7	28	29	3	11	11	6	2	22	28	7	15	0	0	5	12	7	1
Weak (*n* = 3, 3.48%)	9	4	3	3	8	0	3	2	4	4	9	2	9	1	0	8	0	1	1
Clinical isolates																			
Wound (*n* = 21, 24.41%)	15	19	18	20	9	4	10	6	5	13	19	6	14	4	1	3	5	11	4
Urine (*n* = 15, 17.44%)	9	11	14	12	11	14	7	2	1	14	15	1	1	2	0	9	5	5	2
Blood (*n* = 29, 33.72%)	12	3	24	27	2	1	3	11	9	21	24	10	17	6	0	17	8	6	5
Medical devices (*n* = 11, 12.79%)	8	1	3	4	0	0	2	0	0	2	3	0	0	0	0	0	1	1	0
Gender																			
Male (*n* = 19, 22.09%)	19	11	19	13	11	7	6	7	13	17	19	10	8	4	1	17	6	13	5
Female (*n* = 67, 77.90%)	25	22	40	48	11	12	15	12	2	33	42	7	24	8	0	12	13	9	6
Hospital sections																			
Maternity unit (*n* = 6, 6.97%)	6	3	4	6	5	1	3	0	0	5	6	1	2	0	1	2	0	2	0
Pediatrics (*n* = 14, 16.27%)	3	1	11	11	2	3	1	1	2	3	10	2	4	0	0	2	0	1	0
Internal unit (*n* = 13, 15.11%)	6	1	5	9	3	1	1	1	1	1	7	1	3	1	0	1	2	1	1
Emergency (*n* = 17, 8.13%)	10	9	11	13	1	3	6	3	2	11	10	1	7	0	0	7	1	1	1
ICU (*n* = 15, 17.44%)	7	11	13	9	5	9	2	3	1	15	15	3	5	7	0	6	7	7	3
Burns unit (*n* = 14, 16.27%)	12	8	14	13	6	4	8	11	9	14	13	9	13	4	0	11	9	10	6
Neurosurgery (*n* = 7, 8.13%)	0	0	1	0	0	0	0	0	0	1	0	0	0	0	0	0	0	0	0

IMI: imipenem; MER: meropenem; GEN: gentamycin; CIP: ciprofloxacin; ATM: aztreonam; CPE: cefepime; C: colistin; AMK: amikacin; TMP/SMX: trimethoprim/sulfamethoxazole; ICU: Intensive Care Unit.

**Table 3:** Correlation between efflux pumps, biofilm formation, separated clinical isolates, and antibiotic resistance in P. *aeruginosa*.

Biofilm	Efflux pump families						
	RND types			MFS types			SMR types
	*MexA*	*mexB*	*oprM*	*oprD*	*tetA*	*tetR*	*emrE*
Strong	*p* = 0.009	*p* = 0.001	*p* = 0.008	*p* = 0.009	*p* = 0.039	*p* = 0.022	*p* = 0.041
Moderate	*p* = 0.017	*p* = 0.050	*p* = 0.036	*p* = 0.002	*p* = 0.075	*p* = 0.033	*p* = 0.019
Weak	*p* = 0.043	*p* = 0.003	*p* = 0.032	*p* = 0.017	*p* = 0.015	*p* = 0.048	*p* = 0.028

Clinical isolates							
Wound	*p* = 0.040	*p* = 0.029	*p* = 0.016	*p* = 0.048	*p* = 0.066	*p* = 0.043	*p* = 0.051
Urine	*p* = 0.056	*p* = 0.037	*p* = 0.044	*p* = 0.011	*p* = 0.006	*p* = 0.005	*p* = 0.006
Blood	*p* = 0.250	*p* = 0.089	*p* = 0.014	*p* = 0.097	*p* = 0.25	*p* = 0.1	*p* = 0.068
Medical devices	*p* = 0.058	*p* = 0.060	*p* = 0.084	*p* = 0.12	*p* = 0.09	*p* = 0.049	*p* = 0.17

Gender							
Male	*p* = 0.31	*p* = 0.11	*p* = 0.33	*p* = 0.47	*p* = 0.075	*p* = 0.082	*p* = 0.059
Female	0.085	0.020	0.009	0.005	0.066	0.043	0.051
Hospital sections							
Maternity unit	*p* = 0.049	*p* = 0.016	*p* = 0.055	*p* = 0.005	*p* = 0.009	*p* = 0.045	*p* = 0.043
Pediatrics	*p* = 0.019	*p* = 0.062	*p* = 0.035	*p* = 0.015	*p* = 0.009	*p* = 0.45	*p* = 0.21
Internal unit	*p* = 0.31	*p* = 0.11	*p* = 0.33	*p* = 0.47	*p* = 0.075	*p* = 0.082	*p* = 0.059
Emergency	0.025	0.015	0.084	0.001	0.075	0.033	0.019
ICU	*p* = 0.002	*p* = 0.001	*p* = 0.072	*p* = 0.050	*p* = 0.039	*p* = 0.044	*p* = 0.011
Burns unit	*p* = 0.019	*p* = 0.020	*p* = 0.048	*p* = 0.053	*p* = 0.036	*p* = 0.050	*p* = 0.018
Neurosurgery	*p* = 0.094	*p* = 0.31	*p* = 0.19	*p* = 0.020	*p* = 0.62	*p* = 0. 27	*p* = 0.093

## Data Availability

The data that support the findings of this study are available from the corresponding author upon reasonable request.
